# Non-Conventional Yeasts with Probiotic Potential: Diversity, Functional Role, Sustainable Bioprocessing and Local Relevance

**DOI:** 10.3390/microorganisms14092075

**Published:** 2026-09-17

**Authors:** Izlia J. Arroyo-Maya, Itzel Gaytán, Arlette Santacruz, Sylvie Le Borgne

**Affiliations:** 1 Departamento de Procesos y Tecnología, Universidad Autónoma Metropolitana-Unidad Cuajimalpa, Ciudad de México 05348, Mexico; iarroyo@cua.uam.mx (I.J.A.-M.); igaytan@cua.uam.mx (I.G.); 2 Departamento de Bioingeniería, Escuela de Ingeniería y Ciencias, Tecnológico de Monterrey, Monterrey 64849, Mexico; asantacruz@tec.mx

**Keywords:** non-conventional yeasts, potential probiotic yeasts, *Kluyveromyces marxianus*, *Debaryomyces hansenii*, *Pichia kudriavzevii*, probiotic status, safety, encapsulation, functional foods

## Abstract

*Saccharomyces boulardii* is the most clinically established probiotic yeast, but non-*Saccharomyces* strains also exhibit probiotic potential. These non-conventional yeasts can survive gastrointestinal conditions, adhere to intestinal mucosa, and modulate host physiology in human and veterinary contexts. This review examines their diversity, probiotic traits, safety requirements, and applications, emphasizing strain recovery from fermented foods, environmental niches, and local biotopes. *Kluyveromyces marxianus*, *Debaryomyces hansenii*, and *Pichia kudriavzevii* illustrate this potential, showing promising in vitro and animal evidence, technological versatility, and strain-level safety constraints. Finally, we discuss the path to application and commercialization, including sustainable biomass production, encapsulation, and incorporation into food matrices. The transition from promising isolates to commercial products will require standardized strain-level assessments and clinical studies.

## 1. Introduction

Kollath first used the term **probiotic**, applied to human health, in 1953 to describe *active substances essential for healthy development* [1]. The International Scientific Association for Probiotics and Prebiotics (ISAPP) later retained the FAO/WHO definition of probiotics as *live microorganisms which, when administered in adequate amounts, confer a health benefit on the host* [2]. The ISAPP consensus document has established clear criteria for classifying live microorganisms as probiotics, emphasizing that their **health benefits must be demonstrated in humans** and that they must be isolated, identified, characterized at the strain level, and subjected to genomic sequencing as part of the safety assessment. Live microbes present in fermented foods and beverages are not considered probiotics per se because they occur within complex, dynamic microbial communities whose strain composition and stability are not well defined, although such products may contain microorganisms with probiotic potential and represent important reservoirs of yeast diversity [3].

Early pioneers contributed to developing the probiotic concept and tracing the origin of probiotic yeasts and probiotic yeast candidates [4,5]. In 1906, the French pediatrician Tissier discovered that breastfed healthy infants harbored abundant populations of bifidobacteria in their gut and that these bacteria, once isolated, effectively treated diarrheal episodes in infants [4]. Metchnikoff proposed that consuming fermented milk products could promote health and longevity by modulating the gut microbiota through the fermentative activity of lactic acid bacteria. However, he did not recommend prolonged kefir consumption because of its variable microflora and the presence of yeasts that produce ethanol and may favor the growth of pathogenic bacteria [6]. Later, in the 1920s, the French microbiologist Henri Boulard isolated the ***Saccharomyces boulardii*** (hereafter abbreviated as *Sb*) yeast from lychee and mangosteen peels after observing that infusions prepared from the peel of these fruits had a protective effect against cholera in local populations in French Indochina, initiating the era of the first clinically recognized probiotic yeast strain *Sb* CNCM I-745 [7]. Biocodex (France) owns this strain and globally commercializes probiotic preparations based on it under different names by country [8].

This brief historical introduction highlights two central aspects of the development of probiotic microorganisms. First, in addition to fecal microbiota, fermented foods and other natural substrates have also served as important sources of microorganisms with potential probiotic relevance. Second, precise molecular identification of a microorganism and in vitro demonstration of probiotic-associated traits are insufficient to establish probiotic status; a clearly demonstrated health benefit in the target host is mandatory. Today, *Sb* remains the clinically established probiotic yeast [9,10,11,12,13,14]. However, growing attention has turned to **non-conventional yeasts (NCY), i.e., non-*****Saccharomyces*** **yeasts**, as promising sources of new potentially probiotic strains with diverse physiological and technological properties, as addressed in several previous reviews [2,15,16].

Here, we present a narrative review of NCY with probiotic potential, covering their biodiversity, candidate-strain selection, functional and safety assessments, biomass production, encapsulation, and incorporation into food matrices. We provide a perspective on the evidence and requirements needed to advance from a promising yeast candidate to a safe and effective probiotic application. Table 1 summarizes the scope and key messages of previous reviews while highlighting the contribution of the present work.

In addition, we consider local microbial diversity as a source of candidate yeasts for developing locally sourced probiotic cultures and applications. *K. marxianus*, *D. hansenii*, and *P. kudriavzevii* are used as contrasting examples of NCY with different functional potential, safety considerations, and application readiness. These species are examples of NCY that encompass strains with probiotic potential, rather than probiotic species, since probiotic properties and safety must be established at the strain level.

We identified the literature for this narrative review by searching PubMed (National Center for Biotechnology Information, NCBI), Scopus, Google Scholar, and SpringerLink; we used the latter specifically to identify relevant book chapters. The initial searches used the terms (“probiotic” AND “yeast”) or “probiotic yeast”, without date restrictions. We initially used review articles and general book chapters to map the field and identify established concepts, relevant species, and key studies. We then manually curated the retrieved literature, prioritizing original studies on NCY with probiotic potential. Studies with *Sb* and *Saccharomyces cerevisiae* were included when evidence specific to NCY was unavailable or when they provided relevant comparative or contextual information. Targeted searches subsequently combined the initial terms with descriptors including “non-*Saccharomyces* yeast”, “non-conventional yeast”, “fermented foods”, “bioprospecting”, “clinical trials”, “whole-genome sequencing”, “genomics”, “safety”, “antifungal susceptibility”, “QPS”, “encapsulation”, “storage stability”, “food matrix”, “functional food”, “yeast biomass production”, “sustainable bioprocessing”, “agro-industrial residues”, “downstream processing”, “techno-economic assessment”, and “life-cycle assessment”, and relevant species names. We searched Scopus for information on Qualified Presumption of Safety (QPS) status using “QPS” and restricting the source title to the EFSA (European Food Safety Authority) Journal. For the section on *Sb*, we searched for review articles using “*Saccharomyces*” AND “*boulardii*” in the title. We also consulted the extensive work of Lynne V. McFarland, recognizing her longstanding contributions to establishing clinical evidence for *Sb* as a probiotic yeast. We also screened the reference lists of retrieved publications to identify additional relevant sources. Peer-reviewed original articles, reviews, regulatory documents, consensus statements, and selected book chapters were included, considering the type of strain-level evidence provided (in vitro, cell-based, animal, or human). References were retained only when the evidence reported in the original source directly supported the statement for which they were cited. The final literature searches were conducted during the revision process in August 2026.

## 2. General Properties of Yeasts with Probiotic Potential

Yeasts are unicellular fungi for most of their life cycle, undergoing asexual growth predominantly by budding or fission, and do not form their sexual states within or upon a fruiting body [18]. Strongly associated with human civilization since prehistory to produce fermented foods and beverages, yeasts have a long evolutionary history dating back approximately 500 million years and represent diverse fungal lineages in which yeast-like growth forms have evolved independently [19]. More than 2000 species are currently distributed across the Ascomycota and Basidiomycota phyla [20]. The Saccharomycotina and Taphrinomycotina subphyla comprise ~60% and ~3.3% of the species in the Ascomycota, respectively, while the Agaricomycotina, Pucciniomycotina and Ustilaginomycotina comprise ~18%, ~12% and ~3% of the species within the Basidiomycota, respectively [21].

**Figure 1:**
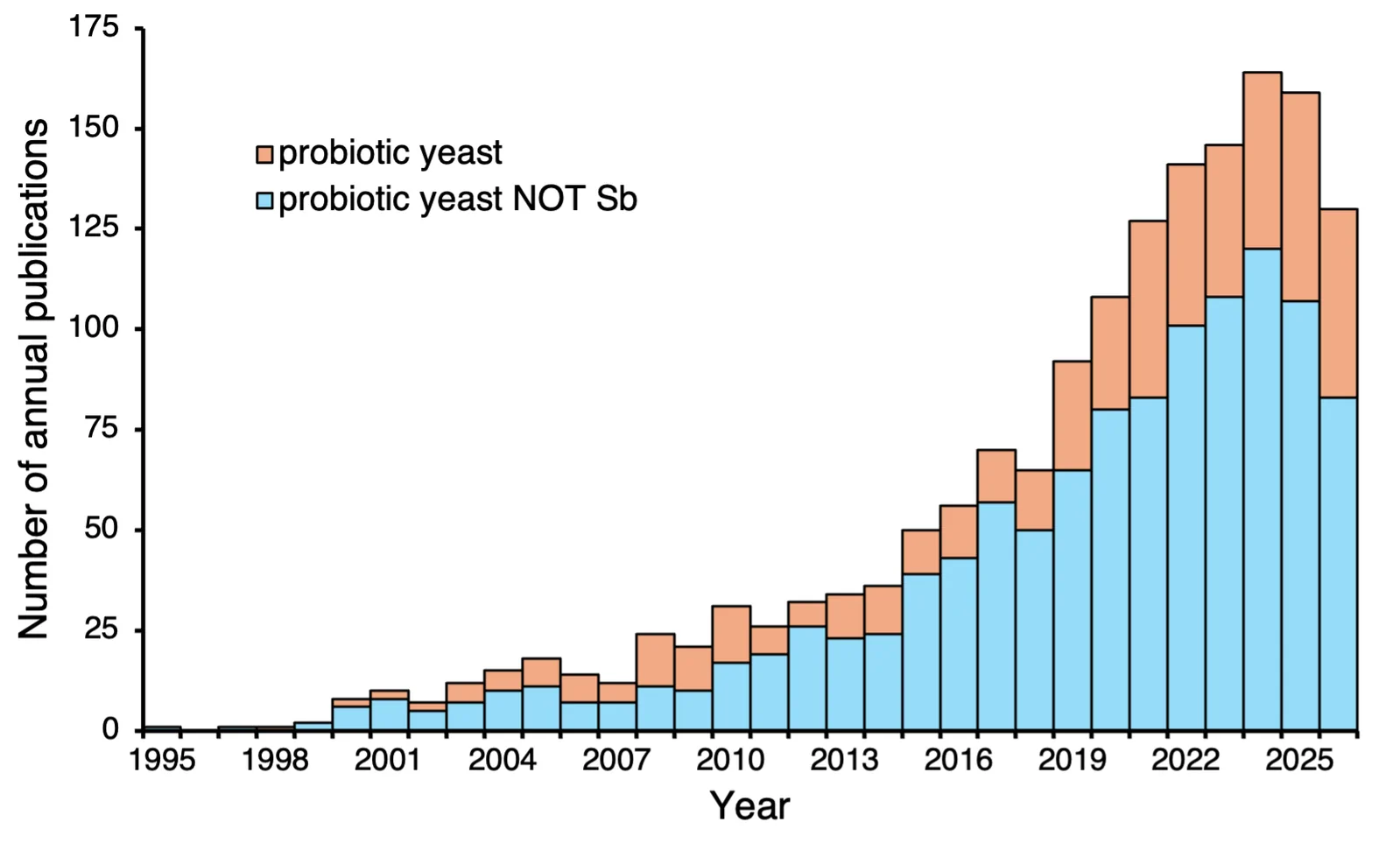
Annual number of publications on probiotic and potentially probiotic yeasts. Retrieved from PubMed using the following queries: ((probiotic[Title/Abstract]) AND (yeast[Title/Abstract])) and ((probiotic[Title/Abstract]) AND (yeast[Title/Abstract]) NOT (*boulardii*[Title/Abstract])). The “Results by year” were then downloaded as a CSV file using the built-in Timeline tool.

Yeasts, mainly *Candida* and *Saccharomyces* (Saccharomycotina), and *Malassezia* (Ustilagomycotina) are normal residents of the healthy human gastrointestinal tract (GIT), where they represent a small fraction (~0.01–0.1%) of the microbiota [22,23]. Because yeast cells are much larger than bacterial cells, their biomass in the GIT may be greater than suggested by this low relative abundance and may influence the activity of health-relevant bacteria [24]. Changes in the gut yeast communities have been associated with gastrointestinal disorders, cancer, and responses to cancer therapy, although causality remains largely unresolved and mycobiome studies have important methodological limitations [24,25]. Over the last two decades, NCY have attracted attention for their probiotic potential in human and veterinary applications [11,24,26,27]. The rising interest in probiotic yeasts is reflected in an increasing number of publications in the last 15 years (Figure 1).

Compared with bacterial probiotics, yeasts differ in several characteristics relevant to their potential use as probiotics, including cell structure, response to antibacterial agents, gastrointestinal behavior, and genetic exchange. Some of these characteristics may confer advantages, though these should not be generalized across yeast species or strains. Yeasts are generally insensitive to antibacterial antibiotics and may therefore remain viable during antibacterial treatment. The transfer of antibiotic-resistance determinants among lactic acid bacteria in fermented foods is a concern that has recently been reviewed [28]. Although less common, **horizontal gene transfer** (HGT) has also been reported in yeasts and should not be considered absent [29]. However, transfer of bacterial antibiotic-resistance genes to yeasts has not been reported. Table 2 summarizes these characteristics, their potential advantages, and the main points to consider when interpreting their probiotic relevance.

**Yeast β-glucans** have a β(1,3) backbone with β(1,6)-linked branches and have shown immunomodulatory activity in preclinical studies; evidence in human studies is more limited [34,40]. Approximately 15–30% of the yeast dry weight is the cell wall, and β-glucans constitute about half of the cell wall’s dry weight [32]. Therefore, administering a probiotic or potentially probiotic yeast is equivalent to delivering a large dose of β-glucans.

Once in the small intestine, particulate yeast β-glucans are absorbed by M cells on the surface of Peyer’s patches. Once engulfed, they are fragmented and act as pathogen-associated molecular patterns (PAMPs) that interact with pattern recognition receptors on immune cell surfaces. This interaction triggers an antimicrobial response and an immune signaling cascade, activating pro-inflammatory cytokines, B cells, and T cells [33]. Yeast β-glucans also induce trained immunity, i.e., the reprogramming of innate immune cells (monocytes and macrophages) via metabolic and epigenetic changes, which promotes enhanced nonspecific protection against subsequent infections by different microbes [34,41].

Trained immunity can also be induced by synthetic ligands derived from bacterial cell walls, such as muramyl dipeptide and its derivatives [42]. These peptidoglycan fragments are difficult to obtain from bacterial lysates and are typically produced by chemical synthesis as non-pyrogenic derivatives for research and specialized therapeutic uses [43,44]. By contrast, yeast β-glucans are safe, abundant structural components of yeast cell walls that are naturally exposed during host–yeast interactions. They are readily purified from yeast cultures and have shown immunomodulatory, antioxidant, and anti-inflammatory effects in different experimental models, including human and mouse cells, with multiple applications in the food and health sectors [40].

## 3. *Saccharomyces boulardii*

**Multiple randomized clinical trials** support the use of *Sb*, especially the CNCM I-745 strain, for which at least 88 trials were reported for 15 different diseases between 1976 and 2009. In addition, pharmacokinetic studies have indicated that this yeast does not persist in the intestines and is completely cleared within 3–5 days after discontinuing treatment [7].

*Sb* has been prescribed for the treatment and prevention of several gastrointestinal conditions, especially those with a predominant inflammatory component, such as pediatric and adult acute diarrhea, inflammatory bowel disease, irritable bowel syndrome, antibiotic-associated diarrhea, *Helicobacter pylori* infections, *Clostridium difficile* infections, traveler’s diarrhea, and enteral feed diarrhea [36]. This yeast has gained increasing attention as a functional ingredient in fermented foods and in foods for special medical purposes [45].

Due to its distinctive phenotypic characteristics, including optimal growth at 37 °C, resistance to low pH and bile salts, acetic acid tolerance, distinct carbohydrate assimilation patterns, and the absence of sporulation [46], *Sb* was initially considered a distinct species within the genus *Saccharomyces*. However, molecular typing studies later indicated that *Sb* isolates are distinct strains of *S. cerevisiae* rather than a distinct species [47], leading to its designation as ***S. cerevisiae*** **var.** ***boulardii*** (referred to as *Sb* throughout this review).

Since the first *Sb* genomes became available, average nucleotide identity analysis and core-genome phylogeny have consistently placed this yeast within the *S. cerevisiae* species, close to *S. cerevisiae* wine strains; however, lineage-specific genomic features have been consistently found in *Sb* commercial strains, such as conserved amino-acid substitutions in SDH1, WHI2, BNA2, and ARO8, an increased copy number of cell-wall mannoproteins, and an increased number of internal repeats within the flocculin genes [48,49,50].

Ref. [51] has extensively reviewed the molecular genetics and probiotic mechanisms of *Sb*, which we briefly summarize below. The substitutions detected in SDH1, which encodes a subunit of succinate dehydrogenase, are thought to be associated with the accumulation of succinate together with the compensatory overproduction of acetic acid through the pyruvate dehydrogenase bypass. Simultaneously, inhibition of acetate consumption could trigger extracellular acetic acid accumulation at 37 °C due to a mutation in WHI2, a negative regulator involved in stress response. These mutations relate to the probiotic efficacy of *Sb*, largely attributed to its **high acetic acid production**, which exerts strong antimicrobial activity. On the other hand, succinate and acetate are both immunomodulatory metabolites that respectively signal pro- and anti-inflammatory responses in the gut, and they are also substrates for colonic anaerobic bacteria that produce SCFAs. Cell-wall mannoproteins are the first line of defense against acetic acid stress, while increased repeats in flocculin genes are associated with strong pathogen binding to yeast, followed by clearance during intestinal transit. Mutations in the BNA2 and ARO8 genes, linked to tryptophan metabolism, may be related to the secretion of tryptophan-derived metabolites that activate aryl hydrocarbon receptor (AhR) signaling, which plays a central role in mucosal immune regulation and epithelial homeostasis. However, these genotype-to-probiotic-phenotype relationships have remained largely inferential, with no loss-of-function validation, and require further studies that integrate high-resolution genomics, targeted metabolomics, and in vivo validation.

*Sb* has several modes of action that target both the host and the pathogenic agents (Table 3). These mechanisms are classified either by biological effect (functional classification) or by site of action (spatial classification): luminal, mucosal, or immune-mediated.

## 4. Isolation Niches and Local Relevance of Candidate Probiotic Yeasts

**Figure 2:**
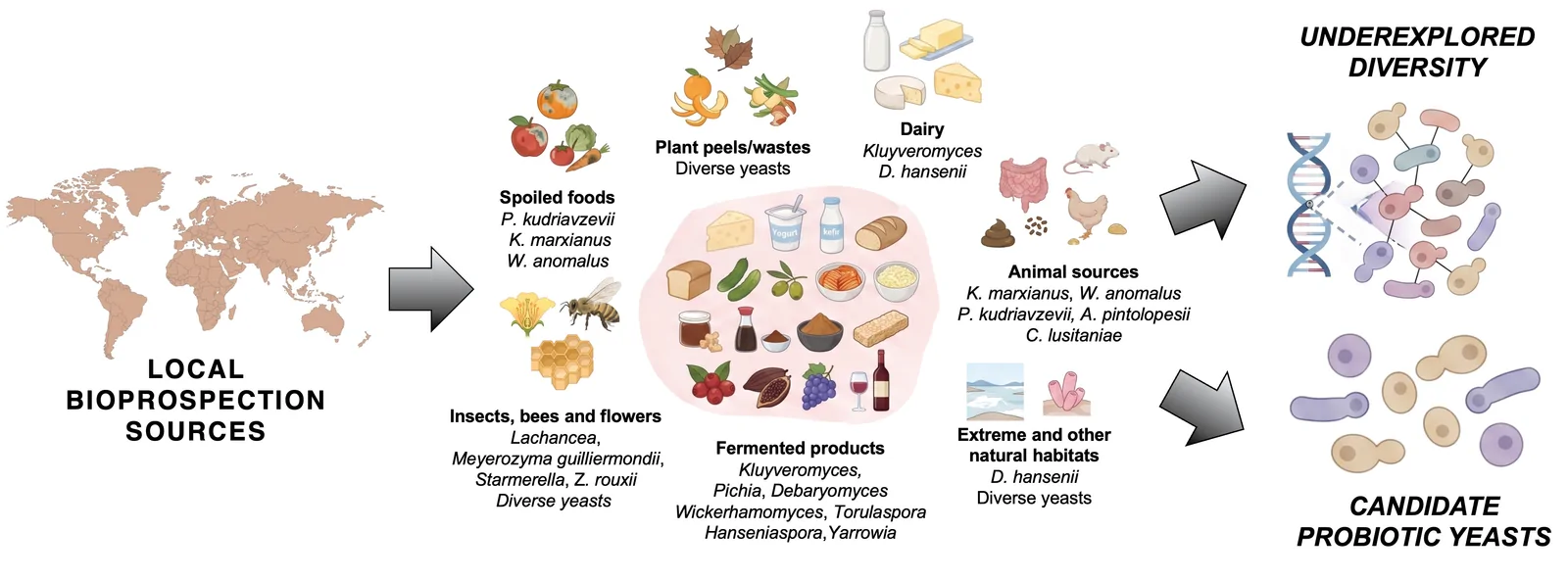
Illustrative overview of bioprospecting sources for isolating NCY with probiotic potential. Created with BioRender. Does not represent original experimental data.

Whole-genome and environmental DNA sequencing has shown that traditional and industrial fermentations already exploit dozens of yeast species beyond *S. cerevisiae* [57]. In contrast, recent reviews have highlighted the probiotic potential of several NCY species [11,13]. The main niches for bioprospecting NCY with probiotic potential are illustrated in Figure 2.

Although it is outside the scope of this work, it should not be forgotten that, in addition to *Sb*, conventional *S. cerevisiae* strains isolated from foods and beverages also exhibit probiotic potential [58]. For example, *S. cerevisiae* C41, an indigenous strain isolated from the Mexican artisanal fermented beverage “Tibicos,” showed higher hydrophobicity, similar auto-aggregation capacity, and resistance to acidic pH and bile compared to *Sb* in vitro [59]. Additionally, several *S. cerevisiae* strains not belonging to the *Sb* lineage are marketed as probiotic yeasts by multinational yeast producers [49].

Although the ISAPP does not classify **fermented products** as probiotics because they do not contain defined strains at an established dose per serving [2], they are invaluable sources for isolating new probiotic yeast candidates and constitute the primary biotope for bioprospecting [11]. In vitro tests have shown that yeasts isolated from fermented products may offer potential health benefits, including a direct cholesterol-reducing effect through absorption onto yeast cells or metabolic assimilation; indirect cholesterol removal via bile salt hydrolysis; production of antioxidants, folate, antihypertensive peptides, and hydrolytic enzymes that assist in the breakdown of food; phytase for the degradation of phytic acid (an antinutritional factor); and γ-aminobutyric acid (GABA) and other amino acids, such as glutamate [60,61,62].

Dairy matrices (kefir, artisanal cheeses, koumiss) frequently yield *Kluyveromyces* species, whereas *Pichia*, *Debaryomyces*, *Wickerhamomyces*, *Torulaspora*, *Hanseniaspora*, and *Yarrowia* are mainly recovered from cereals, olives, brined vegetables, cocoa, coffee, fruit, and alcoholic beverages [27,30,63,64,65,66]. The importance of the isolation source is highlighted. For example, non-fermented lychee peels yielded the highest proportion of yeast isolates with probiotic potential (53%), followed by palm wine (25%), fermented shrimp paste (10%), and rice wine (9%) [67]. Lychee peels harbored the greatest diversity of yeasts, as revealed by ITS (internal transcribed spacer) metabarcoding analysis in this study. Hi-C (chromosome conformation capture) metagenomic characterization of spontaneous beer and cider fermentations has recently shown that ITS metabarcoding and short-read shotgun studies have largely **underestimated yeast diversity in fermented foods** [68], indicating that substantial yeast diversity and functional potential remain unexplored.

Other reservoirs include dry-fermented meats (dominated by *D. hansenii*) [69]; spoiled food products, where *P. kudriavzevii* was detected as a strong candidate compared with *Sb* as a reference strain [64]; flowers and bee-associated ecosystems (*Debaryomyces*, *Hanseniaspora*, *Lachancea*, *Meyerozyma guilliermondii*, *Pichia*, *Torulaspora*, *Starmerella*, *Zygosaccharomyces rouxii*) [70,71,72,73]; marine habitats (the halotolerant *D. hansenii*) [74]; neonatal feces (*K. marxianus*) [75]; infant feces (*Issatchenkia orientalis*, current name: *P. kudriavzevii*) [76]; feces of patients with IBD (*Clavispora lusitaniae*) [77]; as well as fermented broiler chicken excreta (*Wickerhamomyces anomalus*) [78] and mouse feces (*Kazachstania pintolopesii*, current name: *Arxiozyma pintolopesii*) [77].

Several factors support the development of **locally sourced** probiotic microorganisms, including their potential adaptation to regional food matrices and production substrates, opportunities for local technological development, reduced dependence on imported proprietary cultures, increased affordability, and cultural relevance [79]. Importantly, local origin should not be interpreted as evidence of greater biological suitability or efficacy for the local population. Additionally, local probiotics may also align with consumer preferences, as a systematic review of 72 studies across 32 countries (85,348 consumers) found that consumers consistently value foods perceived as natural, locally produced, and culturally authentic [80]. In this sense, several region-specific publications highlight the growing interest in local probiotic yeasts.

In sub-Saharan Africa, yeasts with probiotic potential have been isolated from cereal, milk, and cassava-based fermentations, as well as from palm wine [61,81,82]. Indonesian fermented foods have yielded candidate probiotic yeasts from diverse species exhibiting antimicrobial, antioxidant, and immunomodulatory activities [83]. Likewise, NCY with probiotic potential have been isolated from cocoa fermentations in tropical regions [84]. Among 116 yeasts isolated from Brazilian indigenous fermented foods, cocoa fermentations, and kefir, 36 were tolerant to simulated gastrointestinal conditions, and 15 of them matched or exceeded *Sb* in their adhesion and antioxidant properties [85]. Examination of 46 wild non-*Saccharomyces* yeasts from Brazilian forest flowers and fruits yielded two candidates (*Hanseniaspora osmophila* and *Lachancea thermotolerans*) that outperformed *Sb* in gastrointestinal tolerance and autoaggregation assays [70]. The probiotic potential of NCY isolated from Chilean honeys was evaluated, yielding three yeasts (*Z. rouxii*, *Schizosaccharomyces pombe*, and *Metschnikowia chrysoperlae*) that maintained counts of 10^6^ CFU/mL after in vitro digestion [86].

**Figure 3:**
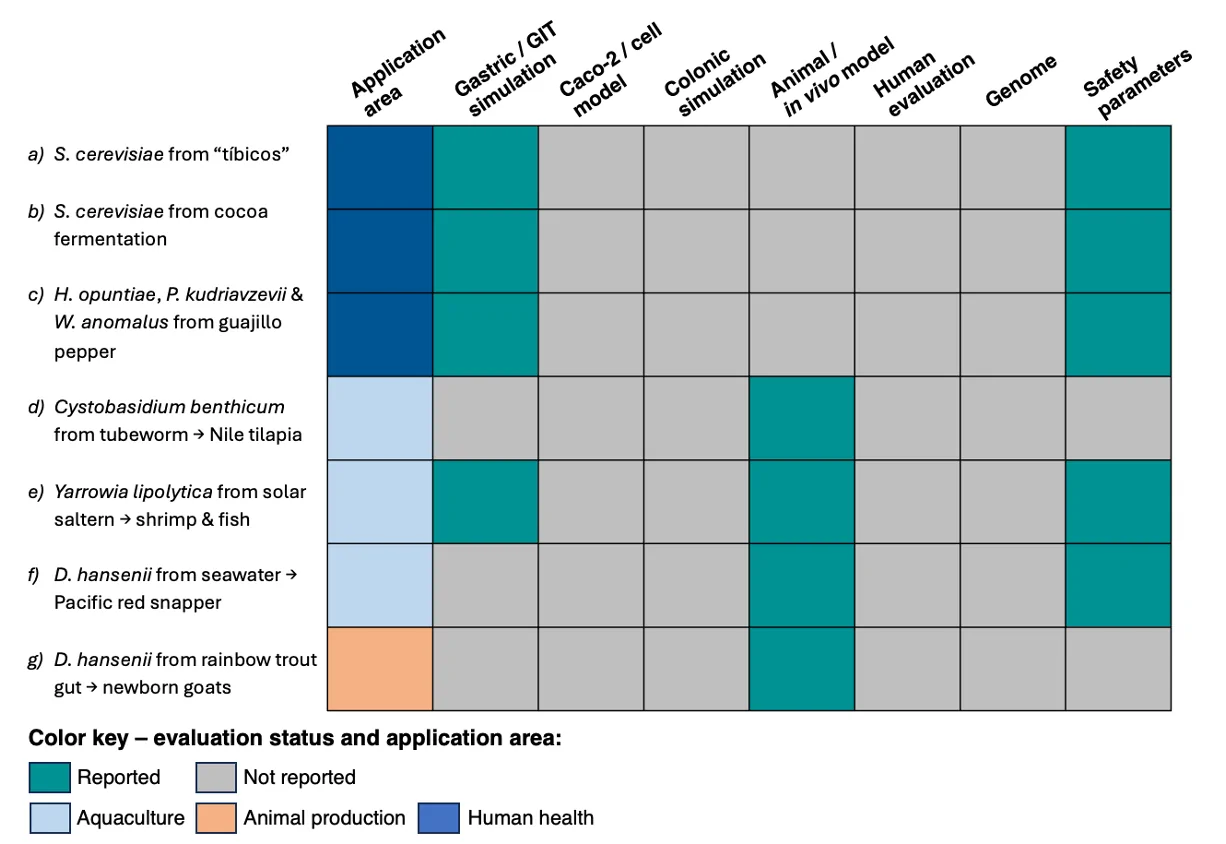
Research carried out in Mexico by different research groups on *Saccharomyces* and NCY with probiotic potential. The figure summarizes previously published studies: (a) [59]; (b) [89]; (c) [90]; (d) [91]; (e) [92,93]; (f) [94]; (g) [95].

Mexico has a long tradition of spontaneous fermentations, which represent a rich source of microbial diversity and an interesting resource for the bioprospecting of strains with probiotic potential and other biotechnological applications [87,88]. Figure 3 shows that research in Mexico has followed two distinct lines of work. The first focuses on *S. cerevisiae* and NCY recovered from emblematic fermentations (cocoa-bean, “tíbicos,” and guajillo-pepper), and includes no evaluation of adhesion to epithelial cells (Caco-2), colonic simulation, whole-genome sequences, or human evaluation. The second is more applied, using marine yeasts for applications in aquaculture and animal production, including animal trials [59,89,90,91,92,93,94,95].

## 5. Snapshot of Three Species with Promising Potential

The probiotic yeast market is dominated by *Sb*. Among NCY with probiotic potential, *K. marxianus*, *D. hansenii*, and *P. kudriavzevii* have received increasing attention and offer contrasting examples of probiotic application potential [13]. These three Ascomycota species combine probiotic-associated properties with physiological and technological traits relevant to food and biotechnological applications [69,96,97,98,99]. However, **available probiotic evidence varies considerably among species and strains**, particularly regarding in vivo validation, safety, and progress toward application. Table 4 compares the three species using selected strain-level examples, including in vitro functional properties, animal and human evidence, safety and regulatory status, and commercial readiness.

*K. marxianus* is a food-grade member of the Saccharomycetales order, often isolated from dairy products, especially kefir, as well as from a variety of other habitats. It is characterized by rapid growth, thermotolerance, and a broad range of carbon sources for growth, including xylose and lactose, and has several current and future biotechnological applications [100]. *D. hansenii* belongs to the Serinales order (within the Pichiomycetes), is recovered mainly from soil, cheese, marine and other salty environments, and has applications in the food industry and in green biotechnology [99,101,102]. *P. kudriavzevii* is classified in the Pichiales order (also within the Pichiomycetes). It is widely distributed in the environment, frequently isolated from traditional ferments, multi-stress resistant, and has applications in the food and biotechnology industries [96].

**Table 4:** Comparison of *K. marxianus*, *D. hansenii*, and *P. kudriavzevii* based on selected strain-level examples.

Species	Key In Vitro Functional and Host-Related Models	Animal Evidence	Safety Characterization/Genomics	Evidence in Target Host	Commercial Readiness/Health Claim
*K. marxianus*	Strain PCH397 (yak milk): high GIT survival (78–99%), hydrophobicity (~81%) and autoaggregation (96%) [103]. Strain A4 (kefir): higher GIT survival than *Sb*, with hydrophobicity, biofilm formation, and autoaggregation [104]. Strain B0399 (dairy): high Caco-2 adhesion; modulation of inflammatory responses in PBMCs/Caco-2 cells; increased bifidobacteria in a human colonic model [105]. Strain CBS 1553: anti-inflammatory Foxp3+ Treg response, contrasting with the TH1 response induced by *Sb* [106].	Strain CIDCA 8154 (kefir) reduced colitis histopathology and circulating IL-6 in mice [107].	QPS status (Qualification 1) ^&^. Strains A4 and A5 (kefir): non-hemolytic; safety confirmed in mice [108]. Draft genome available for B0399 [109].	No controlled human trials identified.	Strong food/industry association. B0399 marketed mainly in Italy as DiarYeast^®^ (“probiotic dairy yeast”).
*D. hansenii*	23 food/fish-gut strains: GIT survival, Caco-2/mucin adhesion and strain-dependent anti-inflammatory responses; some strains showed stronger adhesion or higher IL-10/IL-12 ratios than *Sb* [74].	Enhanced immune/antioxidant responses, growth, gut condition and host defense in gilthead seabream [110,111]; stimulated innate immune and antioxidant parameters in newborn goats [95].	QPS status (Qualification 1) ^&^. May be a less common human pathogen than previously thought because of earlier misidentification [112].	No controlled human trials identified. Probiotic effects reported in terrestrial and aquatic target animals [113].	Potential adoption in aquaculture has been proposed [113].
*P. kudriavzevii*	Strain YGM091 (goat milk): high acid/bile survival, hydrophobicity, aggregation and antioxidant activity; fluconazole resistant, susceptible to other tested antifungals, non-hemolytic and lacking tested virulence enzymes [114]. Strain Y33 (mango pickle): high acid/bile survival, autoaggregation, and cholesterol assimilation; no antifungal testing [115]. Strain 5S5: selected among 105 isolates for GIT survival, intestinal-cell adhesion and hydrophobicity, using *Sb* as reference [116].	Strain YGM091: in vivo safety in *Galleria mellonella* [114].	Not QPS-recommended. It exhibits intrinsic fluconazole resistance and is included in the WHO fungal priority pathogens list [74]. Complete genome available for strain SJP-SNU (fermented plants); no antifungal testing reported; no mortality induced in chicken embryos [117].	No controlled human trials identified. Isolate 8 (rumen fistula of Hu sheep) improved growth, digestibility and rumen fermentation in Hu sheep [118].	No established live human probiotic application identified; rigorous strain-level safety assessment required.

^&^ Qualification 1: absence of resistance to antimycotics used for medical treatment of yeast infections when viable cells are added to the food or feed chain. Section 6 provides a more detailed discussion of QPS status. Abbreviations: GIT, gastrointestinal tract; PBMCs, peripheral blood mononuclear cells; QPS, Qualified Presumption of Safety; *Sb*, *Saccharomyces boulardii*.

Regarding probiotic potential, the strain-level examples presented in Table 4 show promising properties in core in vitro tests (GIT survival, adhesion, antioxidant and immunomodulatory activity), with performance comparable to *Sb* in at least one characteristic, though some differences are observed. For example, *K. marxianus* CBS1553 induced dendritic cell cytokine levels statistically indistinguishable from those of the *Sb* reference strain, indicating immunomodulatory properties in cell-based assays [106], while the kefir isolate *K. marxianus* A4 showed superior performance to *Sb* in simulated GIT conditions, hydrophobicity, biofilm, and autoaggregation [104]. A dairy strain of *D. hansenii* DI 09 adhered more strongly to Caco-2 cells (which mimic intestinal epithelial cells) and to mucin (the intestinal mucus) than *Sb* [74], while several *P. kudriavzevii* isolates from traditional Turkish fermented foods outcompeted *Sb* at acidic pH and in bile [116].

Animal models provide evidence for *K. marxianus* in mice and chicken broilers [104,107,119,120]; for *D. hansenii* in fish and goats [93,94,95,110,111]; and for *P. kudriavzevii* in the invertebrate model host *G. mellonella* and sheep [114,118]. *K. marxianus* B0399 has been evaluated in a human colonic model, showing modulation of both the immune response and the composition of gut microbiota [105]. However, **none of the three species have multiple randomized clinical trials in humans**.

The key differences among the strains discussed from these three species primarily relate to safety and market readiness rather than probiotic potential (Table 4). *K. marxianus* is the most developed species, having a long history of use in dairy applications [100]. Specifically, *K. marxianus* B0399 has an available draft genome [109] and is commercialized in Italy under the name DiarYeast^®^ as a *lactic yeast with probiotic action* [121]. Several strains of *D. hansenii* show promise for use in aquaculture and animal feed, supported by substantial evidence of immunomodulatory effects, modulation of gut microbiota, enhanced cell proliferation and differentiation, and improved digestive functions in both aquatic and terrestrial animals [113].

Although some strains of *P. kudriavzevii* have shown promising probiotic-associated properties in vitro, their use as live probiotics raises safety concerns. Population genomic analyses have clearly demonstrated that *P. kudriavzevii*, an important industrial yeast, and *Candida krusei* (current name: *P. kudriavzevii*), an opportunistic pathogen, are the same species, and that clinical and environmental isolates (including food, fermented foods, and agricultural sources) are not clearly separated into distinct phylogenetic lineages [122]. This point is further supported by Ref. [123], which also reported genetic links between clinical and fermented food isolates. Interestingly, clinical isolates generally showed greater filamentation and biofilm-forming ability, both considered virulence-associated traits, while isolates from fermented foods showed lower susceptibility to several antifungal drugs. Therefore, **food origin alone cannot indicate safety**.

*P. kudriavzevii* is included in the medium-priority group, the lowest of the three priority categories of the WHO fungal priority pathogens list [124], and is intrinsically resistant to fluconazole. Although invasive infections are uncommon, they can be associated with high mortality in vulnerable patients [125]. In this context, **rigorous strain-level safety assessment**, including antifungal susceptibility, is needed before considering *P. kudriavzevii* for use as a live probiotic. Some authors suggest that azoles used in agriculture and food production could select for resistant strains that may reach humans and therefore require further investigation and surveillance [123]. A more restrictive approach proposes using non-pathogenic related species as alternatives for biotechnological and food applications [122]. Another option is to consider the functional properties of *P. kudriavzevii* in non-viable alternatives for postbiotic applications, for example as extracellular vesicles that possess antimicrobial and immunomodulatory properties [126].

## 6. Probiotic Activity and Safety Assessment

Evaluation of the probiotic potential of microorganisms involves a battery of in vitro, in vivo, genetic, and omics-based approaches to assess functionality, safety, and efficacy [127]. These evidence types are not equivalent. In vitro characteristics such as tolerance to the normal human body temperature (37 °C), survival under GIT conditions, adhesion, aggregation, or antimicrobial activity are useful for the initial screening of potential candidates but do not demonstrate probiotic status, which includes *defined contents, appropriate viable count at end of shelf life and suitable evidence for health benefits* and must be *safe for their intended use* [2]. Among these characteristics, the ability to confer a health benefit to the target host is fundamental, and, for intended use in humans, at least one human trial is needed [128]. In this sense, in vitro tests aimed at evaluating health-related benefits for cardiovascular diseases (bile salt hydrolysis, cholesterol degradation, and anti-ACE activity), cancer, lactose intolerance, and antioxidant and oxalate-degrading activity are not sufficient evidence.

As reviewed in Ref. [31], research on NCY probiotic candidates has primarily focused on **in vitro assays** evaluating growth at 37 °C, tolerance to simulated GIT conditions (acidic/alkaline pH, digestive enzymes, and bile), and the ability to adhere to epithelial cells, as well as hydrophobicity and autoaggregation. As shown in Table 4 for the three representative species discussed in this review, the types and extent of tests performed vary considerably among strains, while evidence from animal models and controlled human studies remains limited.

**Figure 4:**
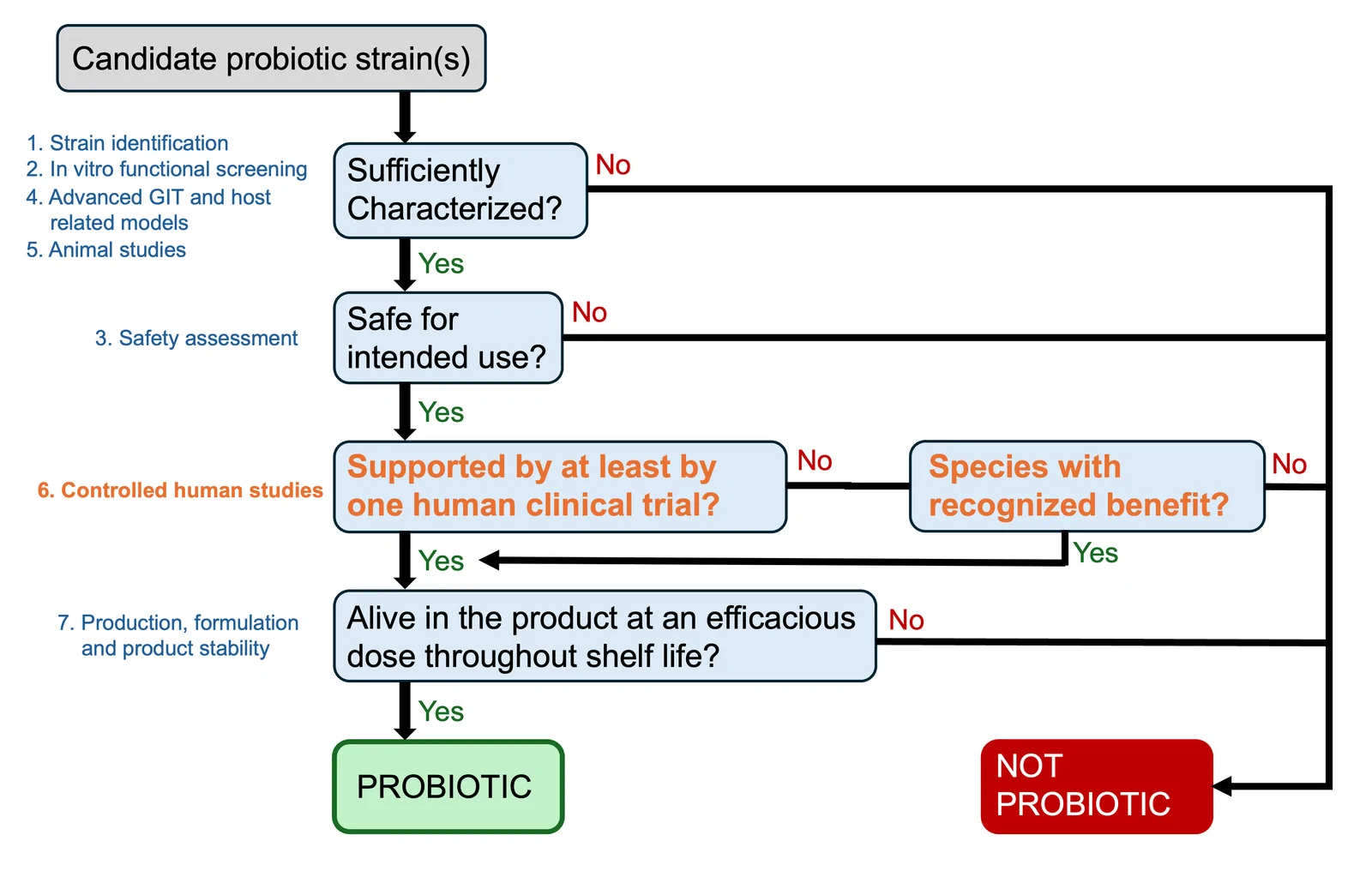
Conceptual decision tree for the assessment of candidate probiotic strains. Adapted from [128]. Numbers correspond to the evaluation approaches summarized in Table 5 and do not indicate a mandatory sequential order.

Probiotic candidate evaluation can be viewed as a progression from strain identification and in vitro screening to initial safety characterization, advanced gastrointestinal and host-related models, animal studies, and controlled human studies (Table 5 and Figure 4). However, this scientific evaluation framework (Table 5) may not be strictly linear, as different types of evidence support a series of decisions (Figure 4) regarding a candidate’s probiotic status according to the four criteria proposed in Ref. [128]. Following these four criteria, a probiotic yeast should be *(i) sufficiently characterized; (ii) safe for the intended use; (iii) supported by at least one positive human clinical trial conducted according to generally accepted scientific standards; and (iv) alive in sufficient numbers in the product at an efficacious dose throughout shelf life* [128].

Within this framework, correct strain identification is the basis, followed by in vitro screening, advanced GIT and host-related models, and animal studies that progressively strengthen the functional evidence supporting candidate selection; however, they do not substitute for demonstrating a health benefit to the host. Safety assessment combines species- and strain-specific information and may require genomic, phenotypic, and host-related evidence, depending on the candidate and its intended use. Table 5 summarizes the main scientific approaches used to evaluate probiotic potential in yeasts, but it does not mean all assays are necessary for every candidate.

As comprehensively reviewed in Ref. [129], laboratory results may not align with the stability and large-scale performance required for industrial applications. These authors recommend a broader dual-screening strategy in which candidate strains are evaluated through two parallel tracks: one integrating safety assessment with viability under GIT and processing-related stresses, and the other evaluating functional properties. Early strain selection may include tests to assess tolerance to relevant production stresses, such as thermal, oxidative, osmotic, and desiccation stresses. However, early selection based on stability may exclude functionally promising strains with poor initial robustness, which could potentially be improved through appropriate production or formulation strategies. Candidates progressing toward commercialization must also meet the regulatory requirements applicable to the intended use and jurisdiction [128,129]. These requirements have been recently reviewed and are not discussed here [130].

**Table 5:** Strain-level assessment of candidate probiotic yeasts. Elaborated using references [31,37,38,131,132,133,134,135].

Evidence Level	Assay or Approach	Purpose
1. Strain identification	Strain-level identification (DNA barcodes/phenotypic tests); WGS and phylogenomic analyses.	Unambiguous taxonomic identification.
2. In vitro functional screening	Simulated GIT survival (acid and bile tolerance/digestion); cell-surface properties (hydrophobicity, auto- and coaggregation); antimicrobial and antioxidant activities; beneficial enzymatic activities.	Identifies promising candidates and provides functional or mechanistic evidence under host-related conditions.
3. Safety assessment	History of safe use; species-specific concerns; QPS status; antifungal susceptibility; hemolysis and other virulence-related phenotypes; genomic screening for virulence- and resistance-associated determinants.	Establishes candidate safety for the intended use.
4. Advanced GIT and host-related models	SHIME or other dynamic GIT models; relevant intestinal or other host–cell models (e.g., Caco-2/HT-29).	Provides physiologically relevant supportive evidence under simplified host conditions.
5. Animal studies	Relevant animal models, including *Caenorhabditis elegans*, *G. mellonella* and mice.	Provides in vivo evidence of biological effects and safety; may also support veterinary or agricultural applications.
6. Controlled human studies	Appropriate study design; defined strain and dose; relevant clinical biomarkers and outcomes.	Demonstrates health benefit in the target host, a key criterion for probiotic status.
7. Production, formulation and product stability	Effective viable dose; survival during production/processing; formulation and shelf-life stability; sensory acceptance.	Determines whether the strain can be delivered at the effective viable dose throughout shelf life.
8. Regulatory assessment and potential application	Requirements according to microorganism, product category, intended use, target population and jurisdiction.	Assesses regulatory requirements for the intended application, including probiotic qualification, health claims and market authorization.

Abbreviations: GIT, gastrointestinal tract; WGS, whole-genome sequencing; SHIME, Simulator of the Human Intestinal Microbial Ecosystem.

Regarding **advanced GIT models**, SHIME (Simulator of the Human Intestinal Microbial Ecosystem) bioreactors have not been widely used to assess the physiology and functionality of NCY probiotic candidates. Although the effects of apple pomace fermented with *Kazachstania barnettii* D1 (current name: *Maudiozyma barnettii*) from pickled beetroot, *Hanseniaspora uvarum* D9 from grape, and *W. anomalus* D11 from wheat flour on the gut microbiota were studied in a multistage SHIME bioreactor, the evaluation focused exclusively on bacterial microbiota dynamics rather than on yeast populations and metabolic activity [136]. Such advanced models can provide in vitro information on candidate activity at the colonic level; however, this type of evidence is only supportive and does not demonstrate efficacy in the host.

The most extensive in vitro studies have been conducted with a collection of twenty yeast strains, including one commercial *Sb* strain, 10 winery-derived *S. cerevisiae* strains, two *P. kudriavzevii* (winery), one *Pichia membranifaciens* (winery), one *L. thermotolerans* (winery), two *H. osmophila* (distillery), one *Candida vini* (winery, current name: *Azymocandida mycoderma*), two *Pichia anomala* (distillery, current name: *W. anomalus*), and one *Zygosaccharomyces bailii* (fermented vegetables) [35,132]. The first set of assays evaluated the strains’ biotechnological and probiotic potential: assimilation of different carbon sources under aerobic and anaerobic conditions, probiotic properties, and attenuation by sonication; the second set of tests addressed safety aspects, including antibiotic resistance tests. NCY often outperformed *Saccharomyces* strains in adhesion to Caco-2 cells (~60–80% versus ~25–45%). However, high adhesion should be interpreted as a candidate-selection or mechanistic trait rather than evidence of probiotic efficacy. Adhesion is controversial because, on the one hand, it facilitates colonization of the intestinal tract, but on the other hand it increases the risk of translocation, especially in immunocompromised patients [127]. Thus, functional and safety interpretations of the same phenotype may differ according to the strain and intended population.

The twenty strains mentioned above clustered into highly correlated groups organized by yeast origin and probiotic properties. Regarding **hemolytic and safety-related enzymatic activities**, none showed coagulase, hemolytic, or DNase activity under the conditions tested, although the absence of these phenotypes alone is insufficient to establish overall strain safety. Half of the yeasts exhibited bile salt deconjugating activity. Bile salt hydrolase activity is also controversial in probiotic evaluation [137]. Deconjugation, i.e., the hydrolysis of the bonds between bile acids and their conjugated amino acids, enhances probiotic microorganisms’ survival in the gut and may contribute to host cholesterol reduction, but excessive activity can have adverse effects on lipid metabolism. On the other hand, degradation of bile salts is also one of the mechanisms used by yeasts to protect themselves against GIT conditions.

Antimicrobial activity against pathogens and susceptibility to clinically relevant antifungal drugs should be clearly distinguished. The former is a potentially beneficial functional property of the candidate yeast, whereas **antifungal susceptibility** is part of its safety assessment. In the study cited above, the tested strains were susceptible to the antimycotics evaluated (nystatin, ciclopirox-olamine, clotrimazole, and fluconazole), and one *H. osmophila* strain was excluded due to excessive biogenic amine production. For candidate probiotic yeasts, antifungal susceptibility is particularly relevant because some yeast species can act as opportunistic pathogens, and safety assessment should consider both known species-level concerns and strain-specific evidence [128]. In contrast, resistance to antibacterial compounds may be technologically or clinically relevant when probiotic yeasts are intended for use during antibiotic treatment, although it should not be interpreted as evidence of probiotic efficacy. Among the antibacterial compounds evaluated, gentamicin and kanamycin have been recommended because yeasts such as *K. marxianus* may show slight sensitivity to these compounds [121].

Regarding **immunomodulatory potential**, 170 yeast strains representing 75 species (mainly Ascomycota and some Basidiomycota) were evaluated in vitro for their ability to modulate cytokine secretion (IL-12, TNF, IL-10, IL-6, and IL-1β) in human dendritic cells [138]. A high diversity of induced cytokine profiles was observed, with clear species distinctions in some genera. For example, *K. marxianus* strains induced cytokine profiles similar to those of *Sb*, whereas the sister species *Kluyveromyces lactis* induced much lower, almost undetectable, levels. *Debaryomyces* strains, by contrast, displayed highly diverse, strain-dependent cytokine profiles. These results illustrate the value of host–cell assays for identifying potentially relevant biological responses, but also the importance of strain specificity. However, they do not constitute evidence of health benefits in the host or clinical efficacy.

Antimicrobial activity against pathogenic microorganisms is a functional property, not a safety criterion. NCY exhibit **antibacterial activity** against several enteropathogens, including the foodborne pathogen *Listeria monocytogenes*. For example, *K. marxianus* and *K. lactis* strains isolated from the traditional French cheese “Tommes d’orchies” inhibited the growth of *L. monocytogenes* in growth inhibition tests on Mueller–Hinton agar [139]. *Pichia kluyveri* CCMA 0615, isolated from cocoa fermentation, strongly co-aggregated with EPEC (enteropathogenic *Escherichia coli*) and *L. monocytogenes*, and outcompeted *Sb* in *L. monocytogenes* exclusion and competition assays during adhesion to Caco-2 cells [140]. An in vitro study evaluated *K. marxianus* isolates from kefir, together with *W. anomalus* and *Pichia manshurica* isolates from grape must, for their ability to protect Caco-2 cells against association and invasion by *Salmonella enterica* subspecies *enterica* serovar Enteritidis [141]. All the tested isolates effectively inhibited *Salmonella* invasion despite not co-aggregating with this bacterium under the conditions tested, illustrating why complementary assays are useful for characterizing functional potential but should not be interpreted individually as evidence of probiotic efficacy.

In addition to antibacterial effects, NCY also exhibit **antifungal activity**. *Issatchenkia occidentalis* strain ApC (current name: *Pichia occidentalis*), which was isolated from fermented apple juice, reduced the adhesion of *Candida albicans* to Caco-2 cell monolayers, suggesting a possible protective potential of NCY against a key virulence trait of this commensal yeast [142]. Overgrowth of the yeast *C. albicans*, a normal gut resident, disrupts intestinal homeostasis and exacerbates inflammatory processes [23].

Safety assessment is an essential component of strain selection. The EFSA **QPS approach** provides a transparent, periodically updated, high-quality scientific basis for evaluating the safety of candidate probiotic microorganisms [128,129]. Microorganisms granted QPS status undergo a species-level evaluation that considers taxonomic identity, the available body of knowledge, history of use, potential pathogenicity, and, where necessary, specific qualifications associated with their intended use. Absence from the **QPS list** does not necessarily indicate that a microorganism is unsafe; rather, it indicates that it requires an independent safety assessment supported by appropriate evidence. Finally, QPS status is neither a product-specific market authorization nor approval of a defined probiotic or therapeutic claim, and it does not override national requirements associated with its intended use.

According to the updated QPS list [143], all yeast species included before June 2026 retain their QPS status. For yeast species intended for use as viable organisms, the EFSA applies “Qualification 1”, which requires the **absence of resistance to clinically relevant antimycotics**. This can be assessed using standardized recommendations, such as the European Committee on Antimicrobial Susceptibility Testing (EUCAST) method for determining minimum inhibitory concentrations of antifungal agents for yeasts [144]. The NCY included in the QPS list with “Qualification 1” are *D. hansenii*, *H. uvarum*, *K. lactis*, *K. marxianus*, *Phaffia rhodozyma*, *Saccharomyces bayanus*, *S. cerevisiae, Saccharomyces pastorianus*, *S. pombe*, and *Z. rouxii* [145]. In contrast, “Qualification 2” applies to *production purposes only*, indicating that viable cells of the production organism should be absent from the final product. The yeasts currently granted this qualification are *W. anomalus* and *Yarrowia lipolytica*. As pointed out in Ref. [128], many microorganisms used in traditional fermented foods and listed in the Inventory of Microbial Food Cultures with Safety Demonstration in Fermented Food Products, updated in 2022 by the International Dairy Federation (IDF) in collaboration with the European Food and Feed Cultures Association (EFFCA), are not included in the QPS list [146].

The ISAPP [2] has recommended **whole-genome sequencing (WGS)** of probiotic microorganisms for precise taxonomic placement. Additionally, WGS is extremely useful for safety assessment. Requirements for WGS analysis to achieve unambiguous taxonomic identification of microorganisms used in the food chain, including yeasts, have been recently published by the European Food Safety Authority (EFSA) [147]. However, unlike bacteria, no workflow currently exists for analyzing safety-related genes in yeasts. This contrast with bacteria largely reflects the complexity of analyzing wild yeast genomes, where ploidy variation, high heterozygosity, and hybrid origins necessitate combining multiple intricate bioinformatic tools [148].

Using CARD (the Comprehensive Antibiotic Resistance Database) and Victors (Virulence Factors database), no **virulence or resistance genes** were detected in the genome of the potentially probiotic strain HJ2 of *P. kudriavzevii* isolated from a marine mangrove [149]. Additionally, the strain showed normal susceptibility to common antifungal drugs as evaluated using the recommended yeast susceptibility tests [144]. Therefore, safety is more clearly demonstrated at the strain level rather than at the species level. Similarly, no resistance genes were found in the genomes of *L. thermotolerans* LT3 and *Saccharomyces uvarum* SERIUS strains with probiotic potential [150]. Finally, even the “human-friendly” yeast *S. cerevisiae* can cause rare opportunistic infections. Comparative phenotypic and phylogenomic analyses have recently indicated that *S. cerevisiae* strains with infective properties, such as pseudohyphal and invasive growth, frequently cluster with industrial baker’s yeast or *Sb* strains and do not form a separate pathogenic lineage [38]. However, the study did not analyze the presence of virulence or antimicrobial resistance determinants in the studied genomes. Moreover, phylogenetic relatedness does not prove pathogenicity. Thus, the study suggests that opportunistic infections may occasionally originate from strains in these lineages in susceptible hosts. These studies reinforce the need for rigorous, strain-level evaluation of both bacterial and yeast potential probiotics as previously indicated in a comprehensive review and meta-analysis [151].

## 7. Sustainable Production of Probiotic Yeast Biomass

A probiotic product must contain viable, well-defined microorganisms delivered at an effective dose and supported by safety assessments and evidence of health benefit [2]. For this reason, the production of yeast biomass intended for probiotic applications cannot be evaluated solely in terms of biomass yield [152]. Substrate selection, cultivation mode, downstream processing, stabilization, and storage should focus on preserving probiotic traits such as stress tolerance, membrane and cell-wall integrity, metabolic activity, storage stability, recovery after rehydration, and survival throughout the entire gastrointestinal passage [15,153,154]. In this context, **probiotic biomass viability** should be evaluated beyond CFU counts, since plate counts do not detect sublethal injury, viable but non-culturable states, or loss of physiological robustness. Flow cytometry, which provides information on membrane integrity, metabolic activity, and physiological damage, seems optimal for probiotic analysis and is applicable to industrial settings [155]. Notably, it has recently been used to evaluate the adhesion of enteropathogenic bacteria to yeasts during in vitro assays [156].

**Fermentation conditions** affect the physiological state of the cells. In *Sb*, biomass produced under different cultivation conditions varied in its ability to recover and grow after passage through sequential simulated GIT conditions, with cells produced in YPD at 37 °C and harvested at the beginning of the stationary phase showing the best performance [153]. Biomass yield and volumetric productivity should be considered together with cultivation time, substrate utilization, and oxygen requirements. As cell density and scale increase, oxygen transfer may become limiting in aerobic yeast production [152]. A recent high-cell-density process for *Sb* evaluated biomass productivity alongside aeration, dissolved oxygen, and feeding conditions, and incorporated a **techno-economic assessment** (TEA) of upstream and downstream operations at a simulated manufacturing scale [154].

**Agro-industrial residues** are attractive substrates because they can link biomass production with side-stream valorization [157,158]. Some NCY can use carbon sources present in residues and biomass hydrolysates and can tolerate relatively harsh cultivation conditions, such as elevated temperatures, high osmolarity, and toxic compounds, although these traits are species- and strain-dependent [159,160]. Biorefinery approaches may improve overall resource efficiency by integrating biomass production with the recovery of additional products. For example, *K. marxianus* biomass from a strain with probiotic potential has been produced alongside bioethanol and whey protein from cheese whey permeate [161], whereas *Sb* biomass has been co-produced with bioethanol using grass juice as an agricultural feedstock [162].

Pretreatment of lignocellulosic biomass may generate toxic compounds, while complex agro-industrial substrates may introduce solids, pigments, proteins, minerals, anti-nutritional compounds, and mycotoxins that complicate biomass recovery, food safety, and downstream processing [163,164,165]. In this context, most studies have focused primarily on biomass yield, while probiotic functionality and toxic compounds from the culture medium have often received limited attention. For example, Se-enriched *S. cerevisiae* biomass was successfully produced from corn and soybean bran hydrolysates [164], while *Sb* was cultivated on parboiled rice effluent [166]. However, the authors do not discuss the complex composition of these media, which includes high concentrations of proteins, minerals, or tannins, and their possible effects on biomass quality and safety. **Contaminant carryover** is especially relevant when the biomass is intended for probiotic applications. Quality assessment should therefore not rely solely on CFU counts and may need complementary indicators of physiological damage or cell integrity [155].

**Figure 5:**
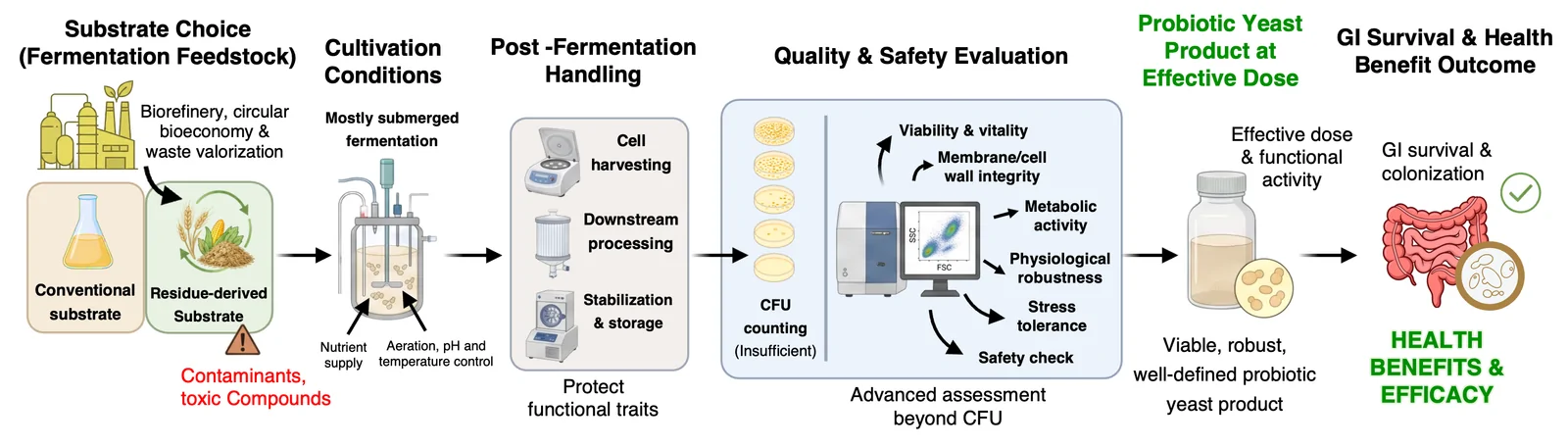
Conceptual overview of considerations for the sustainable production of probiotic yeasts and NCY with probiotic potential. This figure does not represent original experimental data.

Using residue-derived substrates for fermentation does not automatically ensure process sustainability and probiotic functionality, and numerous trade-offs exist across the value chain, including energy and water use, environmental load, downstream losses, physiological robustness, storage stability, and point-of-consumption performance (Figure 5). Pretreatment may also require additional energy, water, chemicals, and separation steps. Economic and environmental evaluations are less developed. The need for additional TEA and **life-cycle analysis** (LCA) of yeast biomass production using different feedstocks has been highlighted [152]. The production of single-cell proteins from *Candida utilis* (current name: *Williopsis jadinii*) biomass using wheat straw as the feedstock, including pretreatment, enzymatic hydrolysis, fermentation, and downstream processing, was evaluated using a TEA approach [167]. It was concluded that plant capacity, investment, and raw material costs were the most critical components. Although this work was not focused on probiotic applications, it shows how strongly feedstock, pretreatment, and downstream operations can affect process feasibility. Together with the TEA available for *Sb* [154], this suggests that sustainability claims for NCY with probiotic potential should be supported by assessments that consider the complete process rather than the substrate alone. Integrated LCA and TEA remain a clear gap, particularly for residue-based production.

## 8. Encapsulation and Controlled Release

Yeasts do not form highly resistant endospores and are more sensitive to environmental stresses than spore-forming bacteria used in probiotic applications [168]. In both *Saccharomyces* and NCY, the cell wall—whose composition, organization, and biophysical properties are tightly regulated in response to oxidative, osmotic, heat, acidic pH, and organic acid stresses—plays a key role [169]. Although yeasts are highly resistant to the acidic pH of the stomach [170] and to industrial stresses in general [171], formulation strategies may be required to preserve their viability during processing, storage, food incorporation, and gastrointestinal transit.

**Figure 6:**
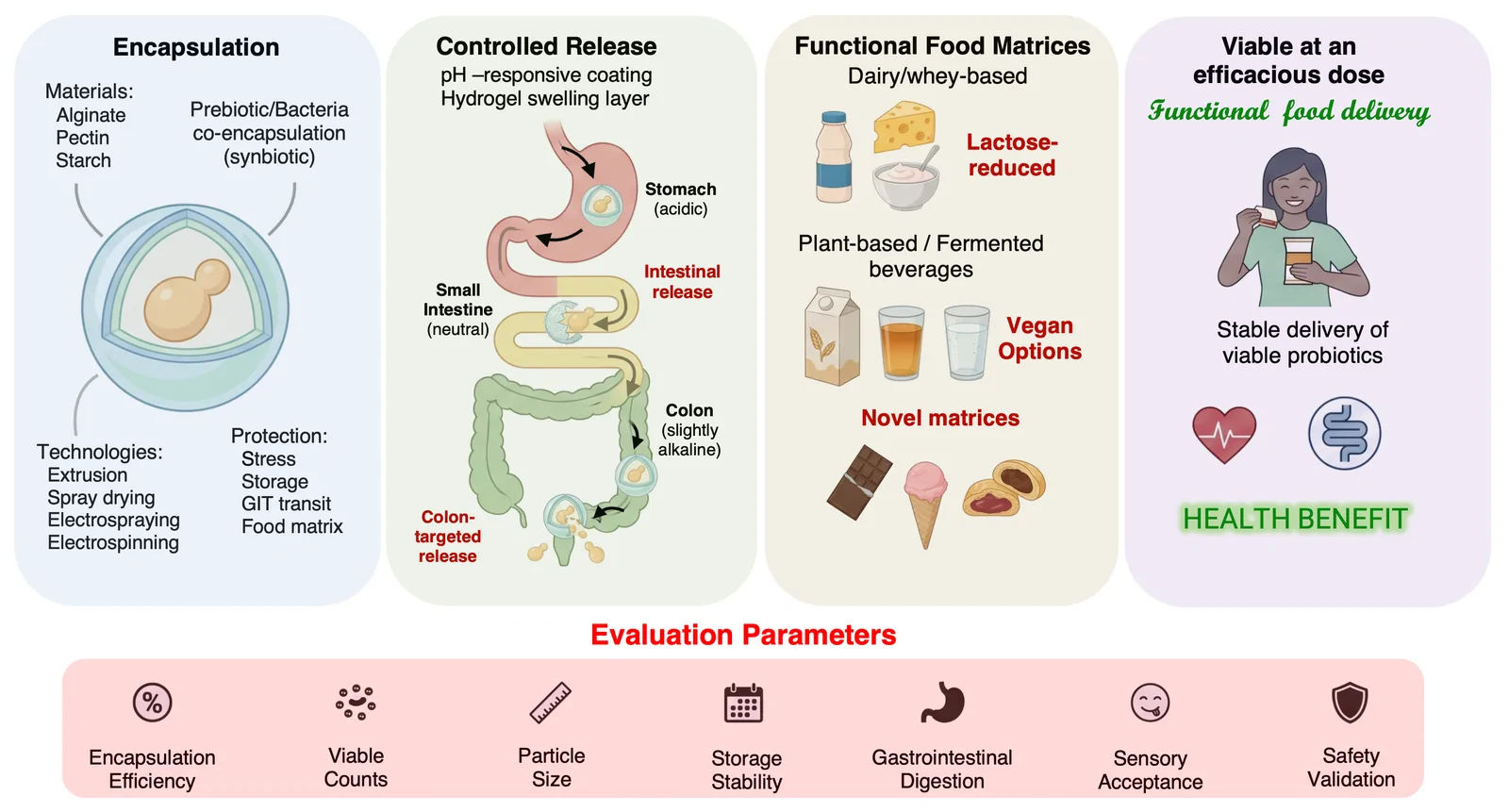
Conceptual overview of the encapsulation of probiotic and potentially probiotic yeasts: from discovery to incorporation into functional foods. Created with BioRender. Does not represent original experimental data.

Encapsulation can provide an additional barrier to regulate release and support the transition of NCY from promising isolates to functional food ingredients (Figure 6). A recent systematic review and meta-analysis of encapsulation of yeast-based probiotics, mainly *Sb*, reported survival rates ranging from 1.7% to 100%, depending on the technique, materials, and stress conditions evaluated [14]. Material selection should prioritize food-grade, biodegradable, and sustainable polymers aligned with circular bioeconomy principles, such as polysaccharides, which are especially attractive because they can form hydrogels, films, beads, coatings, and polyelectrolyte complexes.

Encapsulation research on probiotic yeasts and yeasts with probiotic potential has remained largely focused on *Sb* and *S. cerevisiae*, whereas NCY have received less attention [14,172]. For NCY, strain-specific optimization is necessary because differences in cell size, cell-wall composition, surface charge, and hydrophobicity may affect encapsulation and release.

**Alginate** is the most widely used material because of its low cost, mild gelation conditions, biocompatibility, and ability to form Ca^2+^-crosslinked hydrogels. These properties minimize processing injury, but because alginate beads are highly porous, acids or bile salts can diffuse into the matrix. This material is therefore frequently combined with prebiotics, proteins, chitosan, starch, or cellulose derivatives to improve mechanical strength, reduce porosity, and modulate intestinal release [14,173,174]. Encapsulation of *Sb* in a matrix of cocoa, Na-alginate, and fructooligosaccharides (10:1:1 ratio) allowed for retention of 2.51 log CFU/g more yeast than in non-encapsulated samples after 120 days of storage at 25 °C [175]. Moreover, under simulated gastric conditions, encapsulation reduced viability losses from 6.42 to 3.99 log CFU/g.

**Pectin** is recovered from citrus peel, apple pomace, and other agro-industrial by-products. Low-methoxyl pectin can form Ca^2+^-mediated gels, and its susceptibility to pectinolytic enzymes makes it suitable for colon-targeted delivery and release. Ferulated pectins and arabinoxylans have been used to form electrosprayed microbeads containing viable *Sb* cells, supporting the use of dietary-fiber materials for yeast entrapment [176]. **Cornstarch** has been used to encapsulate *P. kudriavzevii* and *S. cerevisiae* strains with alginate, yielding encapsulation efficiencies of ~90% and survival rates exceeding 90% and 80% under acidic pH and bile salt exposure [177].

**Protein-based materials** offer gelation, emulsification, film formation, water binding, and interfacial stabilization. **Whey protein** concentrate has been used to encapsulate *Sb* in spray-drying studies compared with several other materials (gelatin, modified starch, maltodextrin, pea protein isolate, and gum Arabic). Yeast survivability during spray drying was higher at the lower drying temperature (80 versus 125 °C) and did not vary with the material used, whereas resistance to gastric solution was higher at the highest drying temperature [178]. **Plant proteins** (i.e., vegan materials), such as rice proteins mixed with maltodextrins (25:75%, 80 °C, and feed flow rate 0.75 L h^−1^), were used to encapsulate *Sb*, yielding ~80% survivability after drying, 83% in simulated gastric solution at pH 3 during 3 h, and 71.6% after 20 days of storage at 4 °C, showing that these materials are efficient for microencapsulating and preserving yeast [179].

Regarding encapsulation techniques, **extrusion**, also known as ionic gelation, is simple and cost-effective. A yeast-hydrocolloid suspension is extruded into a gelling solution, commonly calcium chloride. The method has been applied to *K. marxianus* and other NCY strains, improving protection under simulated GIT conditions, though bead size can reach the millimeter scale and influence sensory properties in foods [14,174,180,181]. *Sb* was encapsulated by emulsification using alginate, inulin, and *Opuntia ficus-indica* (*nopal* or prickly pear cactus) mucilage, slightly improving yeast survival (76% versus 63% for free yeast) during refrigerated storage [182].

**Spray drying** is the most scalable technology for the fast production of probiotic yeast powders. It produces dry particles; however, thermal and dehydration stresses can reduce viability [178,183]. This technology has been applied to *Sb* [179] and *K. marxianus* VM004 [184], showing that spray drying can preserve yeast survival under simulated GIT conditions and storage at 4 °C and room temperature, respectively, although long-term stability remains strongly dependent on drying conditions and storage environment (refrigeration versus ambient temperature) [185]. Emerging technologies remain less developed. For example, *Saccharomycopsis fibuligera* VIT-MN04 was encapsulated by **electrospinning** in composite nanofibers [186], while **electrospraying** generated charged droplets containing *Sb* that solidified into crosslinked microbeads under mild conditions [176].

**Co-encapsulation with prebiotics** can support synbiotic formulations. Inulin is the best-documented prebiotic directly incorporated into probiotic yeast capsules, as shown by *Sb* encapsulated with alginate, inulin and *O. ficus-indica* mucilage [182]. There is also direct evidence for psyllium (*Plantago ovata*). *K. marxianus* co-encapsulated with *P. ovata* in alginate-cheese whey particles retained viability during refrigerated storage and simulated gastrointestinal exposure while maintaining adhesion to Caco-2 cells [180].

**Co-encapsulation with bacteria** may combine complementary functions, but differences in cell size, oxygen demand, growth, pH tolerance, and storage stability complicate formulation. Multi-layer microcapsules containing *Lactobacillus acidophilus* in the center and *Sb* in the external layer for differential gastrointestinal release have been developed, showing that *Sb* was released directly in simulated gastric fluid, whereas *L. acidophilus* was released during exposure to simulated intestinal fluid [187]. Co-encapsulation with polyphenols, postbiotics, digestive enzymes, or vitamins remains largely unexplored and should be a future research direction.

Current yeast-specific evidence for **controlled release** mainly includes pH-responsive coatings, layer-by-layer assemblies, swelling-controlled hydrogels, and targeted *Sb* formulations. *Sb* coated with chitosan and dextran sulfate layers improved viability after lyophilization and in simulated gastric and intestinal juice [188]. *Sb* loaded onto Eudragit^®^ S12.5-coated pellets prevented acidic release (gastric passage) and promoted alkaline release (small intestine) under simulated conditions [189]. *Sb* release from alginate–agavin–whey protein beads was mainly controlled by swelling and Fickian diffusion, and the beads reached the colon intact [174].

## 9. Incorporation into Functional Foods and Food Matrices

NCY with probiotic potential can contribute to functional foods in several ways, not only through potential probiotic effects, but also by acting as starters in fermented products and by generating postbiotics, prebiotic oligosaccharides, nutritional compounds, sensory attributes, and protective metabolites [190]. In these applications, a major challenge is maintaining strain viability and functionality through processing, storage, and consumption, particularly in acidic, oxygen-exposed, thermally processed, or low-water-activity matrices. Nevertheless, viable counts should not be treated as a universal threshold for probiotic efficacy or as sufficient evidence of health benefit. The effective dose is strain-, matrix-, serving size-, population-, and indication-specific, and should be justified for each proposed application [191]. **Dairy products** remain the main vehicles for probiotic delivery, and several NCY species have been studied, notably *D. hansenii*, *Y. lipolytica*, *K. lactis*, and *K. marxianus* [192].

Selected strains of *K. marxianus* show high β-galactosidase activity and have been investigated for producing low-lactose fermented milks and yogurts [193,194]. Co-fermentation with lactic acid bacteria has also reduced the characteristic “goaty” flavor of goat milk products while improving aroma component digestibility and antioxidant content when tested in a dynamic biomimetic gastrointestinal system in vitro [195,196]. In addition, *K. marxianus* FM09 (isolated from fermented milk) has been explored for its role in cow-milk cheese aroma development at the laboratory scale, with instrumental analysis identifying changes in aroma-related volatile compounds [197]. These results further support the technological relevance of this species in dairy matrices; however, this functionality should be distinguished from validated probiotic efficacy. *D. hansenii* is frequently associated with the ripening of surface-ripened and smear-ripened cheeses. Its halotolerance allows it to grow under the saline conditions found on cheese surfaces, where amino acid catabolism, proteolysis, and lipolysis contribute to texture and aroma development [198]. In a cheese agar model, *D. hansenii* strains from Danish cheese brines consumed amino acids and produced volatile compounds associated with cheese aroma [199]. The inoculation of *D. hansenii* 304, together with other ripening yeasts and bacteria, into pilot-scale Munster-type cheeses showed that this strain grew throughout ripening, contributed to surface deacidification, and was associated with physicochemical changes in the cheese [200]. *D. hansenii* has also been retrieved in fermented sausages and dry-cured meats, where selected strains can enhance aroma formation and inhibit spoilage or toxigenic fungi [201]. Inoculation of *D. hansenii* strains isolated from dry-cured ham into dry-cured pork belly inhibited the growth of Enterobacteriaceae and contributed to changes in texture, free amino acid content, and the production of aroma compounds [202], while *D. hansenii* strains isolated from dry-cured Iberian pork loin exhibited antifungal activity in a meat-derived medium [203]. These results further support the technological relevance of *D. hansenii* in cheese and **meat matrices**; however, these properties should be distinguished from functional effects in the host.

As demand for **vegan** and **lactose-free** products rises, plant-based foods are emerging as carriers for probiotic yeasts. *P. kudriavzevii* M28, isolated from a traditional African cereal-based fermented food, survived under simulated gastrointestinal conditions and adhered to Caco-2 cells. When co-cultured with a strain of *Lactobacillus fermentum* (current name: *Limosilactobacillus fermentum*) in a pearl millet gruel, this strain enhanced folate content [204]. Similarly, *D. hansenii* (isolation source not reported), when co-cultured with *Lactiplantibacillus plantarum* in a plant-based beverage (based on oat, sunflower seeds, and almonds), maintained viability, exhibited survival under simulated GIT conditions, and showed in vitro antioxidant activity [205]. Again, these findings support the functional and technological potential of selected strains in plant-based matrices, but evidence of health benefits in the host remains lacking.

Yeasts are key components of the microbial consortia in kombucha and kefir. These symbiotic cultures of bacteria and yeast (SCOBY) create a complex flavor profile and serve as natural sources of microorganisms with probiotic potential. Species of *Saccharomyces*, *Brettanomyces*, and *Pichia* are commonly found in these fermented products and have been proposed to contribute to the reported health benefits of these drinks [206,207]. However, under the current probiotic definition, fermented beverages cannot be considered probiotic per se because their complex, variable microbiota do not allow each strain to be linked to a demonstrated health benefit in the host at a defined, effective dose.

The inherent robustness of yeasts enables their incorporation into **novel and non-traditional food matrices** that are generally considered challenging for bacterial probiotics. Low water activity provides a protective environment for probiotics. Studies have demonstrated that *Sb* can be incorporated into dark **chocolate** formulations and maintain high viability, especially when stored at refrigerated temperatures [208]. **Ice cream** can serve as a carrier for probiotic yeasts because fat and total solids may provide cryoprotection. *Sb*-enriched ice cream has maintained viable counts within the commonly cited probiotic range of 10^6^–10^7^ CFU/g throughout storage [209,210]. **Baking** compromises probiotic yeast viability; therefore, *Sb* is more effectively added after baking through creams, fillings, or coatings, or protected by microencapsulation. Single- and double-layered *Sb* microcapsules were incorporated into cake fillings and icings after baking, and pre-baking addition was also evaluated [211]. The results support post-baking incorporation and encapsulation. For incorporation into plain cakes before baking, double-layered microencapsulation, with a hydrophobic outer layer fabricated by spray chilling and a hydrophilic inner layer produced by spray drying, increased the survivability of *Sb* cells. However, synbiotic bread formulations with *Sb* remain promising [212].

## 10. Conclusions

NCY from local biotopes are an untapped source of candidate probiotic yeasts. However, they cannot currently substitute *Sb*, which remains the only probiotic yeast with human clinical trials, proven efficacy for several gastrointestinal conditions, and worldwide commercialization. *Sb* strains form a distinct lineage within *S. cerevisiae* with specific genomic markers, possibly related to probiotic behavior. Several NCY species isolated from diverse biotopes, especially fermented foods and beverages, have shown tolerance to GIT conditions, adhesion, antimicrobial activity, antioxidant capacity, and immunomodulatory effects. Nevertheless, their health benefits have not been established in humans.

Yeasts from local traditional fermentations and regional biotopes may be culturally relevant, compatible with regional food matrices, and potentially useful for local production systems that use available substrates or agro-industrial residues, supporting regional economic development. Nevertheless, geographic origin, traditional use, and a long culinary history do not substitute for strain-level evidence of safety and efficacy when a strain is proposed for use in supplements or functional foods. Moreover, although the circular use of agro-industrial residues may support sustainability and regional development, complex substrates must be carefully evaluated because they can affect biomass yield, reproducibility, safety, and final product quality.

*K. marxianus* and *D. hansenii* are food-associated yeasts with technological versatility and promising functional evidence. In contrast, *P. kudriavzevii* should be considered a functionally promising but safety-sensitive species. Although some strains display strong in vitro probiotic traits and technological robustness, their association with clinical isolates imposes a substantially higher safety burden for any live human probiotic application than for generic industrial biotechnology uses. Therefore, promising traits such as stress tolerance, enzyme production, phytase activity, or antagonistic activity should not be interpreted as sufficient evidence of live probiotic suitability.

The roadmap for NCY development should include unambiguous taxonomic placement, whole-genome analysis, antifungal susceptibility testing, evaluation of virulence-associated traits, in vitro functional characterization, relevant animal models, and, ultimately, well-designed controlled human studies. For species with unresolved safety concerns, particularly *P. kudriavzevii*, non-viable alternatives such as postbiotics, extracellular vesicles, enzymes, or purified metabolites may be more realistic and safer than the use of live human probiotics.

Standardized assessment workflows, a stronger understanding of genotype-to-phenotype relationships, and carefully designed clinical trials will be essential for advancing from promising isolates to safe, evidence-based applications in supplements, functional foods, or beverages. In this context, including more NCY species on the QPS list may facilitate future development, but QPS status should not be confused with safety assessment, product-specific authorization, or validated probiotic efficacy.

## 11. Future Perspectives

Many NCY show promising results in in vitro screening tests, but few advance to clinical validation or commercial use. The challenge is to identify candidates and determine the necessary evidence to support their probiotic use. Candidate yeasts need more standardized, strain-specific evaluation, including accurate taxonomy supported by WGS and phylogenomics, safety assessment, and antifungal testing. Cell viability assessments are crucial, especially in complex substrates or food matrices, where traditional biomass measures may not differentiate viable cells from non-cellular components of the matrix. Advanced viability methods should be considered. Combining genomic data with transcriptomic, metabolomic, and phenotypic information can clarify genotype–phenotype links. This should be complemented with physiologically relevant gut models, such as dynamic gastrointestinal systems or organoids, before human trials.

Formulation remains a limitation. Most studies focus on *Sb* and *S. cerevisiae*, while NCY may need strain-specific approaches. Shelf life, functional preservation, and performance in food matrices are underexplored. Selecting candidates requires considering probiotic functionality and stress tolerance during production, processing, and storage. A dual screening of function, safety, and robustness can aid in development. Valuable functional strains should not be discarded due to initial technological weaknesses; process optimization and protective strategies can improve them.

LCA and TEA are essential, including process data on pretreatment, biomass productivity, energy and water use, downstream processing, product quality, and scale-up. While many studies focus on strain selection based on probiotic properties, less is publicly known about the production, stabilization, and formulation of NCY candidates. This is not due to a lack of technology, as the industry has extensive practical experience, but much remains proprietary and underreported in the literature. Collaboration between academia and industry is crucial to connect functional strain selection with scalable production, stabilization, formulation, and product development. Harmonizing regulatory requirements for strain identification, safety, and efficacy is also key to advancing promising NCY from research to validated foods or supplements.

## Figures and Tables

**Table 1:** Scope and contributions of selected reviews and book chapters on probiotic yeasts and yeasts with probiotic potential. The table summarizes information from the cited publications.

Reference	Type of Publication and Main Scope	Key Message	Focus of the Present Review
Vohra and Satyanarayana (2013) [9]; Choudhary et al. (2017) [10]	Book chapters. Probiotic yeasts in human health with focus on *Sb*: mechanisms, clinical applications and safety.	Establishes the clinical and mechanistic basis for *Sb.* Emphasis on strain specificity and safety.	*Sb* as the clinically established probiotic yeast.
Perpetuini et al. (2022) [5]	Book chapter. Overview of probiotic yeasts: taxonomy, *Sb* and its mechanisms of action and health effects, other yeasts with probiotic potential, encapsulation.	Only a limited number of yeast strains are commercially available (*Sb* and *Kluyveromyces marxianus* B0399). Beneficial traits are often strain-specific. Need for thorough characterization of new candidates.	Focuses on NCY candidates: diversity and sources, functional and safety assessment, production and encapsulation, food applications, progression from candidate to probiotic status.
Vergara et al. (2023) [11]	Narrative review. NCY as probiotic candidates: diversity, functional traits, bioactive compounds, food applications, multiomics, and microencapsulation.	Highlights the diversity and promising functional properties of NCY and their potential for functional-food and nutraceutical applications.	Examines how functional evidence, strain-level safety, and technological aspects affect progression toward application.
Shahryari and Sadeghi (2023) [12]	Book chapter. Yeasts with probiotic potential in fermented foods and production of bioactive metabolites.	Shows how candidate probiotic yeasts can contribute to health-related properties as well as food quality, safety, and technological functionality.	Links the value of food-derived yeasts and other traditional sources with the evidence required to progress from a candidate to a probiotic application.
Tullio (2024) [13]	Narrative review. Established, emerging, and next-generation probiotic yeasts * and candidates, including mechanisms, safety, and engineered strains.	Identifies several promising NCY candidates while emphasizing strain specificity and the need for human studies to establish safety and efficacy.	Examines selected NCY candidates across different levels of evidence and relates biological evidence to safety and application readiness.
Oliveira et al. (2024) [14]	Systematic review and meta-analysis of probiotic yeast encapsulation with evidence mainly based on *Sb*.	Shows the potential of encapsulation to improve yeast viability and identifies the need for process standardization.	Places encapsulation within the pathway from candidate selection to formulation and food applications. Exemplifies with NCY.
Moonsamy et al. (2024) [15]	Narrative review. Probiotic yeasts and candidates: lists mechanisms, health benefits and in vitro tests. Presents general concepts for probiotic biomass production, formulation, and delivery.	Integrates health applications with production and formulation requirements and highlights opportunities for conventional and engineered yeasts.	Connects strain-level evidence and safety, sustainable production, food-matrix applications, and regulatory considerations. Exemplifies with NCY.
Cicero et al. (2026) [16]	Narrative review. Comparison of bacterial and yeast probiotics, their distinct mechanisms and gut-health effects, *Sb* and *K. marxianus* as main yeast examples.	Highlights the complementary functional profiles of bacterial and yeast probiotics, proposes a framework for complementary use in intestinal homeostasis support, emphasizes the need for clinical validation.	Sources, diversity and development of NCY candidates, strain-level functional and safety assessment, production, formulation and technological aspects, and food applications, addresses progression from candidate toward probiotic status.
Present review	Narrative review of NCY with probiotic potential, from strain discovery to application.	Integrates functional evidence, strain-level safety, biological efficacy, production aspects, formulation, and food applications.	Comparative analysis of *K. marxianus*, *Debaryomyces hansenii*, and *Pichia kudriavzevii* as contrasting examples to identify gaps and factors that determine progress toward application and commercialization.

* Next-generation probiotics are probiotics from genera with no history of use as probiotics and no established safety record, and they are likely to require the safety, efficacy, manufacturing, and clinical evidence expected for a therapeutic drug [17]. Although NCY with probiotic potential do not necessarily fit the conventional definition of next-generation probiotics, as they are frequently isolated from foods, fermented products, or environmental sources rather than identified from the human microbiome, many share a limited history of use as probiotics and a limited strain-specific body of knowledge. Their development may therefore require similarly rigorous safety, functional, and clinical evidence.

**Table 2:** Probiotic-relevant differences between yeasts and bacteria.

Characteristic	General Comparison	Potential Advantage of Yeasts	Points to Consider
Cell size	Yeast cells are typically larger, although size varies among taxa and growth conditions [9,10,30,31].	Larger cell size may influence interactions with microorganisms and host surfaces.	Cell size alone does not predict adhesion, pathogen exclusion, or probiotic efficacy.
Cell wall	Yeast cell walls contain β-glucans, mannoproteins, and chitin, bacterial cell walls are based on peptidoglycan [32].	β-Glucans and other cell-wall components may contribute to immunomodulatory effects [33,34].	Cell-wall composition and structure vary with species, strain, and physiological state. Biological effects depend on dose and experimental model.
Response to antibiotics	Yeasts are generally insensitive to antibacterial antibiotics, whereas bacterial susceptibility varies according to species, strain, and antimicrobial agent [30,35].	Yeasts may remain viable during antibacterial treatment.	Antifungal susceptibility should be assessed as part of candidate yeast strain-level safety evaluation [35].
Horizontal gene transfer	HGT is well established in bacteria and has also been documented in yeasts [28,29].	Potentially lower risk of acting as vectors for the spread of antibacterial resistance determinants.	HGT should not be considered absent in yeasts, and its potential safety implications require strain-level evaluation.
Gastrointestinal behavior	Yeasts generally have a lower optimal growth pH (approximately 4.5–6.5) than bacteria. Acid and bile tolerance and gastrointestinal persistence are strain dependent. Transient persistence is well documented for *Sb* but should not be generalized to other yeasts [9,10,30,31,36,37].	Tolerance to acidic conditions may favor survival through the stomach and contribute to the delivery of viable cells to different sites of the GIT ^$^, from stomach to colon.	In vitro tolerance to GIT conditions does not demonstrate probiotic efficacy. The relevance of survival and persistence depends on the proposed site and mechanism of action. Transient persistence does not exclude opportunistic infection in susceptible hosts [13,36,38].

^$^ Gastric pH is 1.0–2.5, proximal small intestine pH is 6.6 ± 0.5, and terminal ileum pH is 7.5 ± 0.4, with a sharp fall to 6.4 in the caecum, and a progressive rise to 7.0 ± 0.7 in the left colon [39].

**Table 3:** Summary of *Sb* mechanisms of action. Elaborated from references [52,53,54,55,56].

Functional Classification	Site of Action	Mechanisms
Antitoxin effects	Luminal	Proteolytic degradation of toxins, toxin binding, inhibition of toxin–receptor interaction.
Direct pathogen inhibition/competitive exclusion	Luminal	Binding of pathogens and flushing during intestinal transit, inhibition of pathogen adhesion and invasive properties.
Modulation of normal microbiota	Luminal	Restoration of microbial balance after antibiotic treatment.
Physiologic/barrier protection	Mucosal (epithelial)	Tight junction stabilization, reduced permeability, protection against apoptosis.
Trophic/nutritional effects	Mucosal (epithelial)	Polyamine production, stimulation of enterocyte growth, brush-border digestive enzymes and glucose transport, secretion of invertase.
Metabolic regulation	Luminal and mucosal	Restores SCFAs levels (acetate, propionate, butyrate), improved nutrient absorption.
Immune system regulation	Mucosal and systemic	Modulation of pro-inflammatory cytokines and inflammatory immune cells, IgA stimulation, inhibition of inflammatory signaling pathways NF-κB and MAPK.

Abbreviations: IgA, immunoglobulin A; MAPK, mitogen-activated protein kinase; NF-κB, nuclear factor kappa B; SCFA, short-chain fatty acid.

## Data Availability

No new data were created or analyzed in this study. Data sharing is not applicable to this article.
